# Supplementary material for: Neolithic culinary traditions revealed by cereal, milk and meat lipids in pottery from Scottish crannogs
Source: Nat Commun. 2022 Sep 6;13:5045. doi: 10.1038/s41467-022-32286-0 (PMC9448721; doi:10.1038/s41467-022-32286-0)
Supplement: Supplementary file 1 — Supplementary Information [file 41467_2022_32286_MOESM1_ESM.pdf]

Supplementary Information for

Neolithic culinary traditions revealed by cereal, milk and meat lipids in pottery from Scottish crannogs

Simon Hammann[a,b,*], Rosie R. Bishop[c], Mike Copper[d], Duncan Garrow[e,*] Caitlin Greenwood[a], Lanah Hewson[e,f], Alison Sheridan[g], Fraser Sturt[h,*], Helen L. Whelton[i], Lucy J. E. Cramp[a,*]

a Department of Anthropology and Archaeology, University of Bristol, 43 Woodland Road, Bristol BS81UU, UK

b Department of Chemistry and Pharmacy, Friedrich-Alexander-Universität Erlangen-Nürnberg, Nikolaus-Fiebiger Straße 10, 91058 Erlangen, Germany

c Arkeologisk Museum, Universitetet i Stavanger, Peder Klows gate 31A, 4036 Stavanger, Norway

d School of Archaeological and Forensic Sciences, University of Bradford, Richmond Road, Bradford BD7 1DP, UK

e Department of Archaeology, University of Reading, Whiteknights Box 227, Reading RG6 6AB, UK

f Present address: Museum of London Archaeology (MOLA) Northampton, 30 Billing Road, Northampton NN1 5DQ, UK

g c/o Scottish History & Archaeology Department, National Museums Scotland, Chambers St, Edinburgh EH1 1JF, UK

h Department of Archaeology, University of Southampton, Avenue Campus, Highfield, Southampton SO17 1BF, UK

i Organic Geochemistry Unit, School of Chemistry, University of Bristol, Cantock's Close, Bristol, BS8 1TS, UK

*** corresponding authors:**

Simon Hammann
Email: simon.hammann@fau.de
Phone: +49 9131 8565391

Duncan Garrow
Email: d.j.garrow@reading.ac.uk
Phone: +44 118 3786458

Fraser Sturt
Email: F.Sturt@soton.ac.uk
Phone: +44 238 0599422

Lucy J. E. Cramp
Email: lucy.cramp@bristol.ac.uk
Phone: +44 117 3311210

This pdf contains:

Supplementary Discussion

Supplementary Figures:

Supplementary Figure 1: Scatter plot showing the $\delta^{13}C$ values of $C_{16:0}$ fatty acids plotted against $\Delta^{13}C$ values ($\delta^{13}C_{18:0}$ - $\delta^{13}C_{16:0}$) for the samples from all four crannogs
Supplementary Figure 2: Partial GC-QToF MS chromatograms and GC-QToF MS spectra of sterol oxidation products
Supplementary Figure 3. Partial GC-FID chromatograms showing the performance of the method to enrich cereal biomarkers
Supplementary Figure 4: Bar charts showing the average distribution of different alkylresorcinol (AR) homologues in modern reference samples of bread wheat, spelt, einkorn, emmer, barley and rye.
Supplementary Figure 5. Partial GC-QToF MS Extracted Ion Chromatograms (m/z 268.1315) of reference cereal lipid extracts.
Supplementary Figure 6: Maps of Loch Bhorgastail and Loch Langabhat showing the positions of ceramic and sediment samples analysed
Supplementary Figure 7: Bar charts showing the distributions of C_{20}-C_{34} n-alcohols in lipid extracts from sherds which contained alkylresorcinols
Supplementary Figure 8: Bar charts showing the distributions of C_{12}-C_{30} fatty acids in lipid extracts from sherds which contained alkylresorcinols
Supplementary Figure 9: GC-QTOF MS extracted ion chromatograms of lipid extracts of the three sediment samples from Loch Bhorgastail
Supplementary Figure 10: GC-QTOF MS extracted ion chromatograms of extracts of absorbed residues from samples BHO16-26a, BHO16-26b, and BHO16-9a
Supplementary Figure 11: Bar charts showing the distributions of C20-C34 n-alcohols in lipid extracts from sherds that contained alkylresorcinols and three sediment samples from Loch Bhorgastail

Supplementary Tables:

Supplementary Table 1: Available radiocarbon data for the four sites
Supplementary Table 2: Results from organic residue analysis of the ceramic sherds

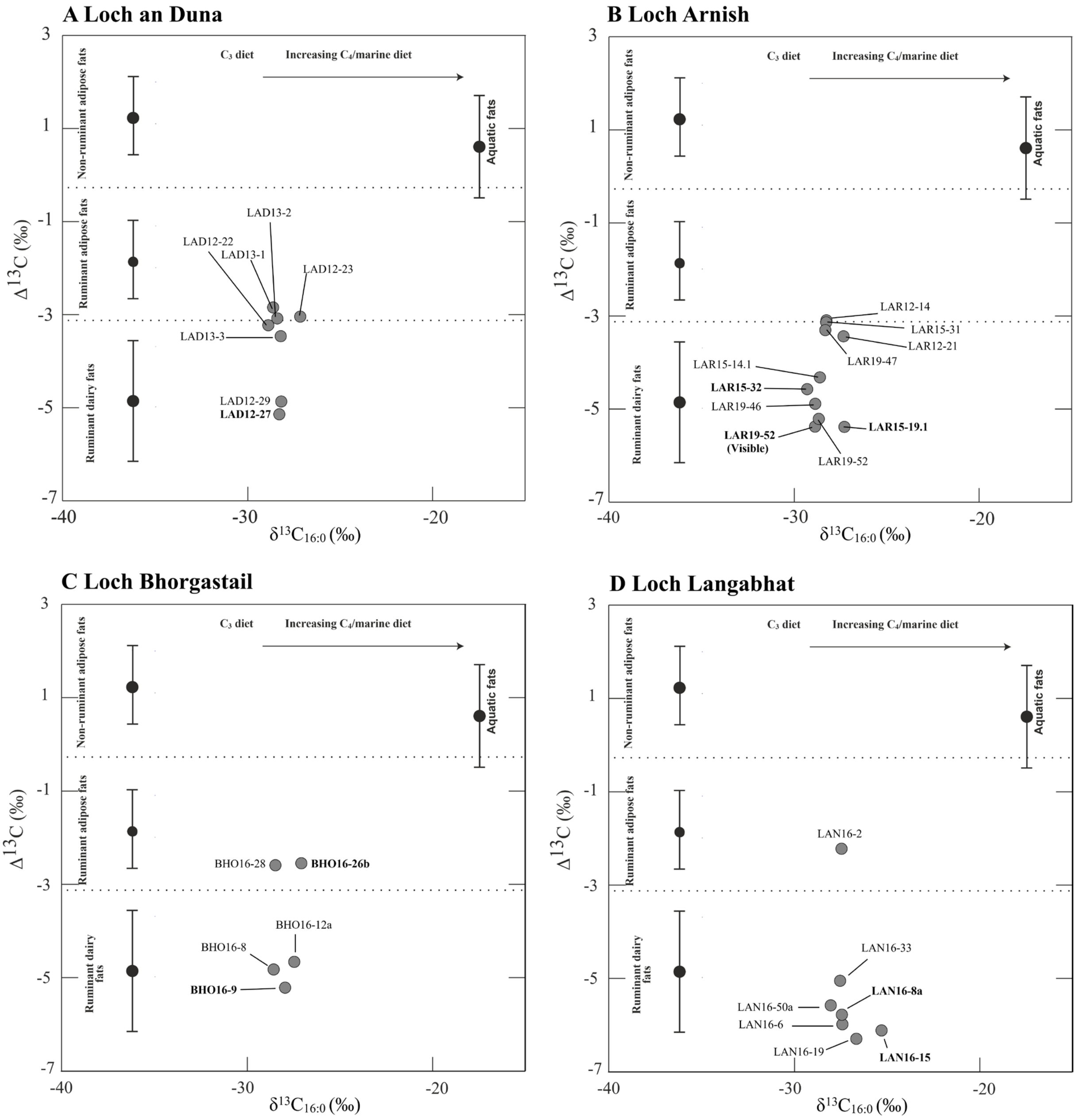

Supplementary Figure 1: Scatter plot showing the δ^{13}C values of $C_{16:0}$ fatty acids plotted against Δ^{13}C values (δ$^{13}C_{18:0}$ - δ$^{13}C_{16:0}$) for the samples from Loch an Duna (A), Loch Arnish (B), Loch Bhorgastail (C) and Loch Langabhat (D). The Δ^{13}C ranges for the modern references for ruminant dairy fats (Δ^{13}C = -4.9±1.3‰), ruminant adipose fats (Δ^{13}C = -1.9±0.9‰), non-ruminant adipose fats (Δ^{13}C = 1.2±0.9‰) and aquatic fats (Δ^{13}C = 0.7±1.0‰) are plotted to the left and right of the diagram as means with one standard deviation. All δ^{13}C values obtained for modern reference terrestrial animal fats were from animals raised on pure C_3 diet [1] and have been adjusted for the post-Industrial Revolution effects of fossil fuel burning, by the addition of 1.2 ‰ [2]. Reference values for aquatic fats are from fish caught in Scandinavian wild waters (marine) and a UK lake and Kazakh river (freshwater fish)[3]. Extracts with cereal biomarkers are labelled in bold. Source data are provided as a Source Data file

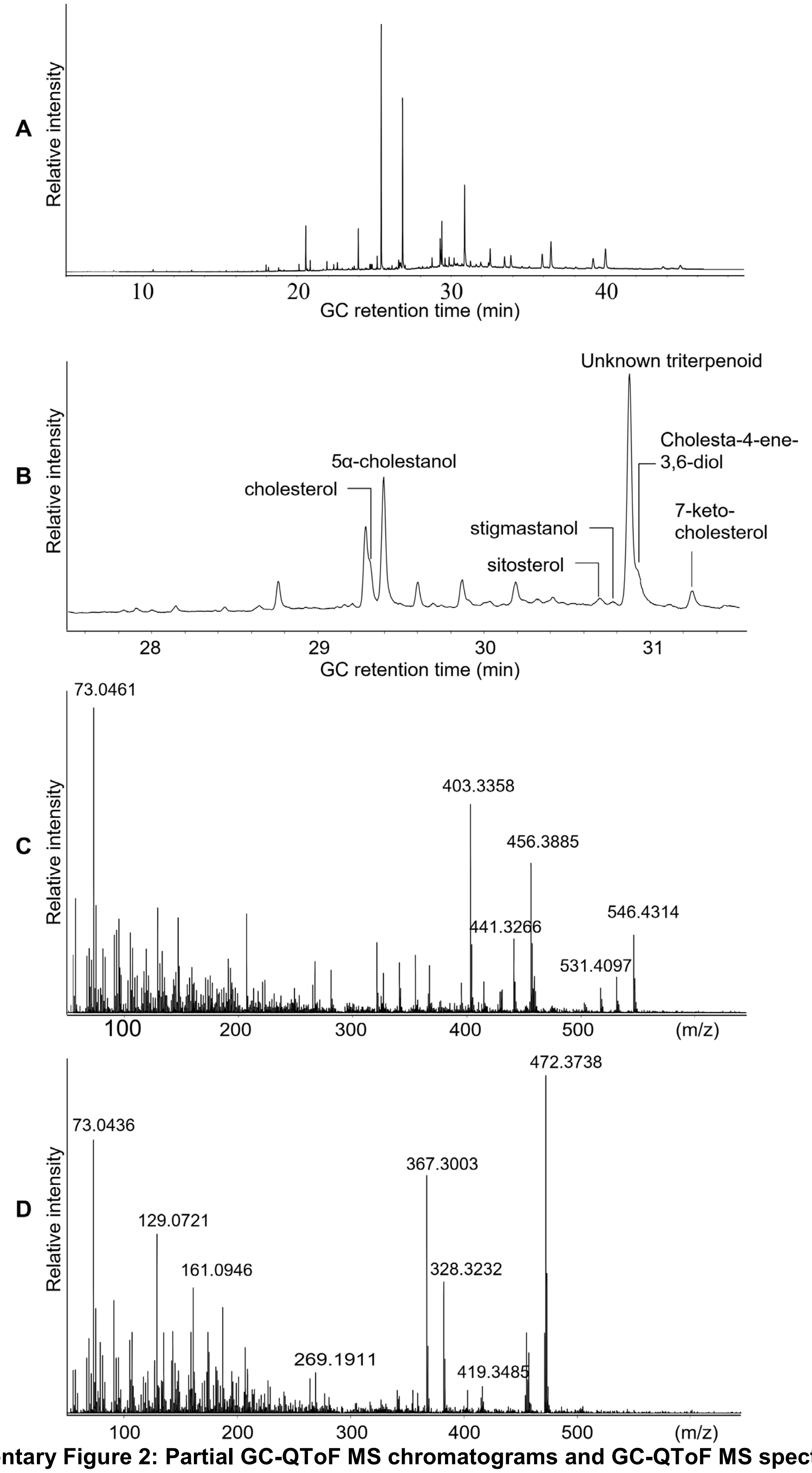

Supplementary Figure 2: Partial GC-QToF MS chromatograms and GC-QToF MS spectra of sterol oxidation products: Partial GC-QToF MS chromatogram of SPE fraction two of sample BHO16-9 after trimethylsilylation (A) and enlarged region between 28 and 31 minutes (B) showing the elution of sterols and sterol oxidation products. The mass spectra of the peaks marked as cholesta-4-ene-3,6-diol (C) and 7-keto-cholesterol (D) in B) are shown below.

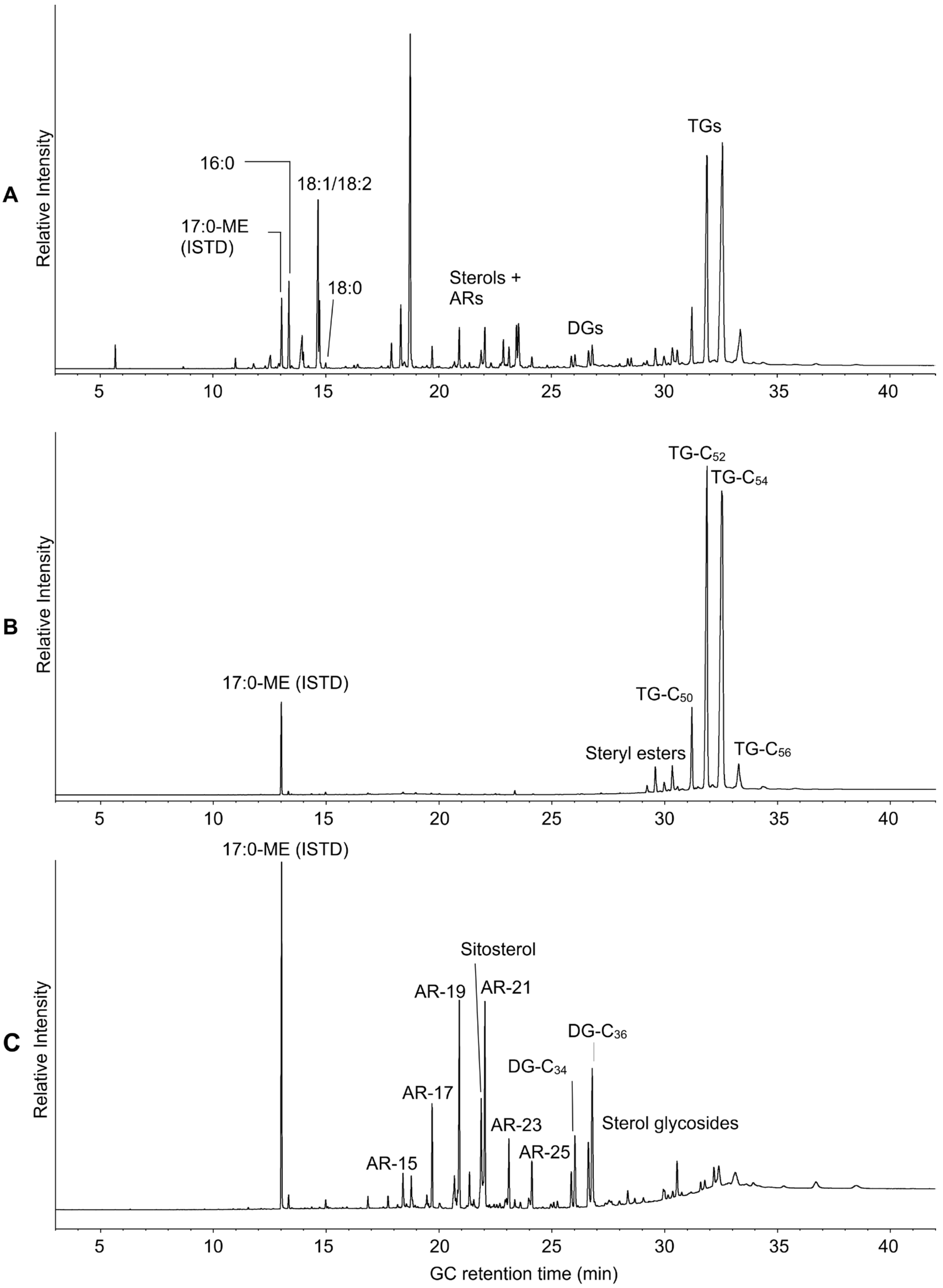

Supplementary Figure 3: Partial GC-FID chromatograms showing the performance of the method to enrich cereal biomarkers. GC chromatograms of a trimethylsilylated reference cereal lipid extract (A) and aminopropyl SPE fractions 1 and 2 (B,C, after silylation) showing the elution of cereal biomarkers in fraction 2. Peaks labelled with C_x-TG and C_x-DAG are triacylglycerols and diaclyglycerols with a total number of X carbons in the fatty acid chains, respectively. The peak marked with ISTD is an internal standard which was added to all fraction before injection at the same concentration.

Supplementary Discussion

Source specificity of alkylresorcinol patterns in archaeological samples

It is well established, that the distribution of AR homologues differs between modern cereal species and can be used as an analytical tool for discrimination. [4-6]. Modern varieties of all relevant *Triticum* species feature AR-21 as most abundant homologue, while the dominant alkylresorcinol homologue in (modern) barley varieties, which is the only other cereal crop relevant for our study region, is AR-25 (**Supplementary Figure 4)**. AR-25 could not be detected in any of the samples, only AR-21 and AR-23.

Previous research on the chemical analysis of residues in a well-preserved Bronze Age container from Switzerland - microscopically identified to consist largely of wheat – provided a distribution of AR homologues very similar to modern wheat [7]. This suggests that the AR composition of cereals has not changed drastically over time and distributions in modern cereals could be used for comparison. Furthermore, in reference experiments involving the cooking of cereals in replica pots it could be seen that the AR pattern absorbed into the ceramic matrix was largely retained, with a slight shift towards longer chain homologues [8]. Similarly, we found that during degradation under anoxic conditions (as existed at our sites) the original AR pattern was still retained, while under oxic conditions shorter chain ARs were preferentially lost (**Supplementary Figure 5**). It therefore seems most likely that the alkylresorcinol pattern present in the residues in this study indicates the processing of some *Triticum* species, i.e. einkorn, emmer, spelt or bread wheat.

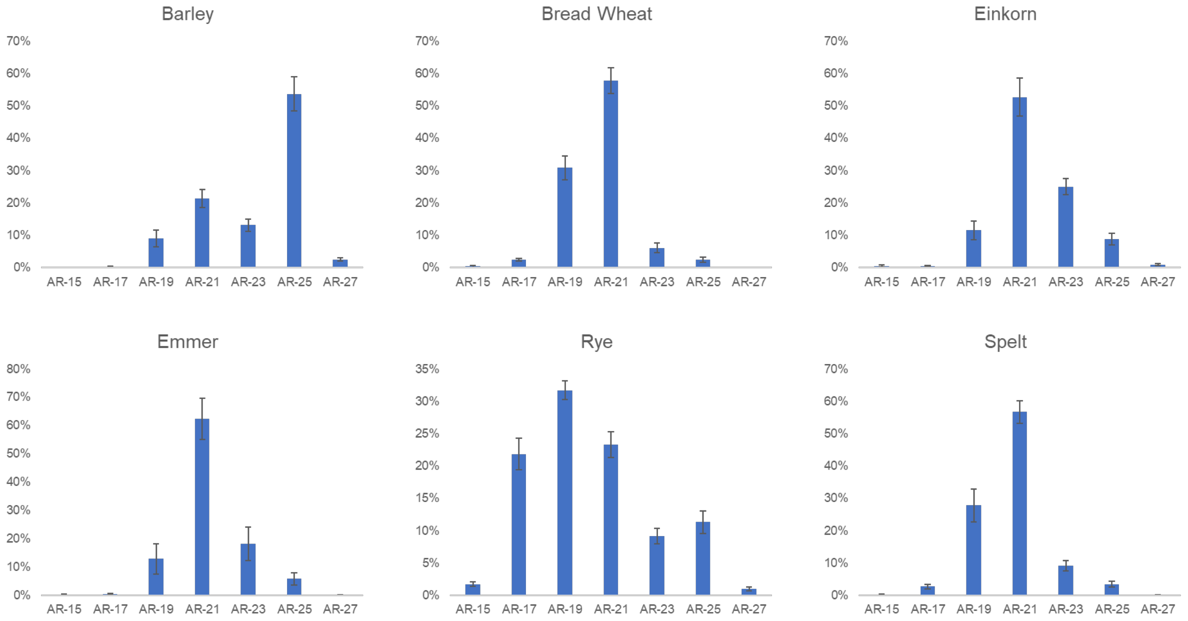

Supplementary Figure 4: Bar charts showing the average distribution of different alkylresorcinol (AR) homologues in modern reference samples of bread wheat, spelt, einkorn, emmer, barley and rye. Displayed are mean values and error bars show standard deviations. Data from [6]. Source data are provided as a Source Data file.

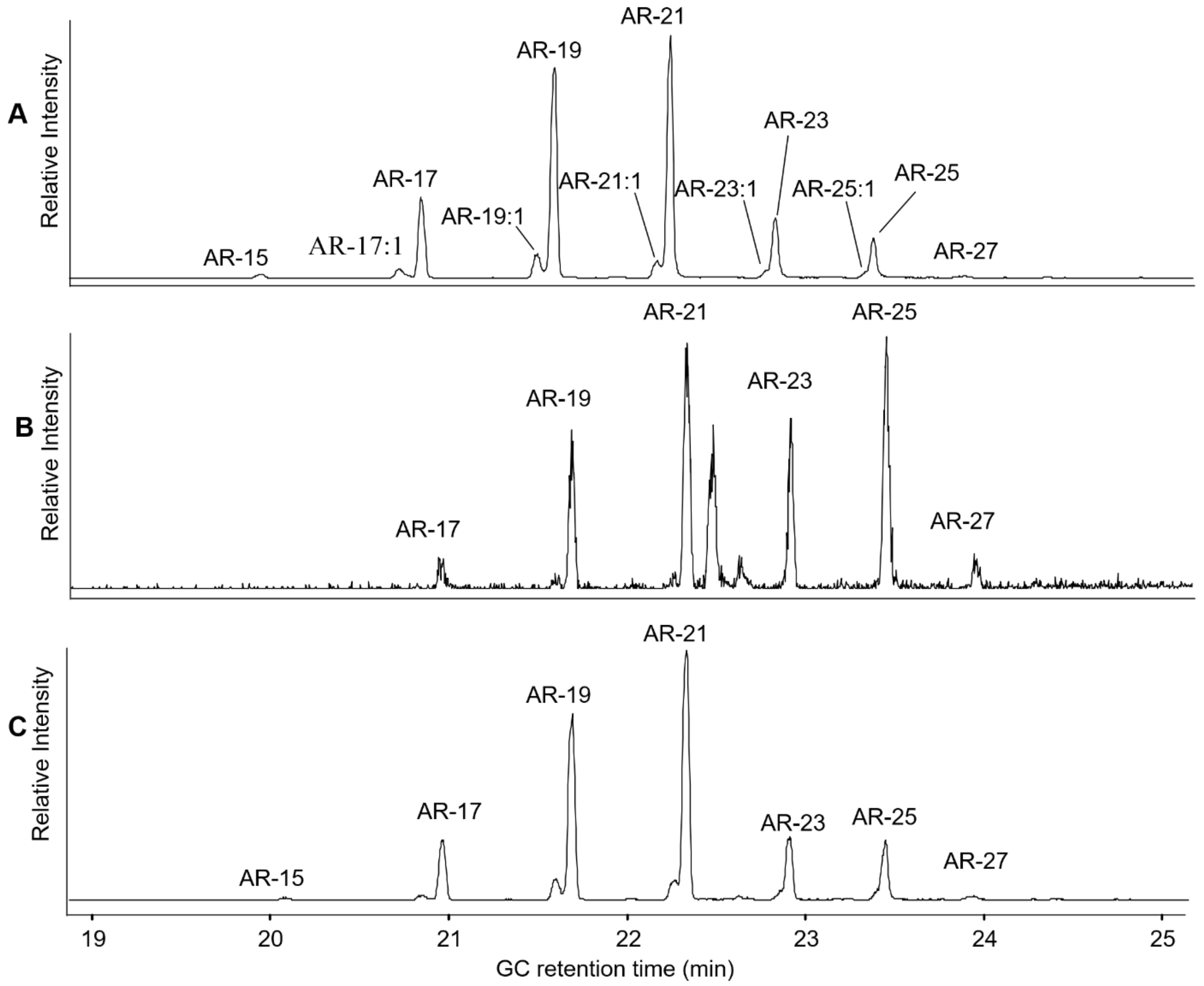

Supplementary Figure 5: Partial GC-QToF MS Extracted Ion Chromatograms (*m/z* 268.1315) of reference cereal lipid extracts. The Extracted Ion Chromatograms show the elution of trimethylsilylated alkylresorcinols (AR-X:Y, with X carbons and Y double bonds in their alkyl chain) in a cereal lipid extract (A) and extracted lipids from reference sherds that were dosed with this extract after 20 weeks of simulated microbial degradation under oxic (B) or anoxic conditions (C). For full description of the experiment and instrumental parameters see [8].

Alkylresorcinols as potential environmental signals

Since ARs can be found in sedges and sedge-derived peats [9,10], it is essential to explore, and discount, a possible environmental origin for the ARs identified in the pottery here. While our samples were not found deposited in peat, possible peat incursion cannot be ruled out over the course of the last 5500 years (since there is considerable run-off of water into these lochs from the local peat-rich landscape on each), which raises the question of whether an uptake of ARs into the sherds might have occurred. We therefore conducted a series of experiments, including analysis of lipid-extracted sediments from the loch bed, as well as additional analyses of the inner and outer surfaces of sherds.

We believe that an environmental signature is highly unlikely as the source of the ARs we identified in the crannog pottery, due to the following conditions that could be reasonably expected for this explanation, **not** being met:

1) **ARs would need to be relatively mobile in watery environments**. Due to their hydrophobic structure ARs are poorly water soluble, and our own experiments showed that even in boiling water only low quantities of alkylresorcinols were liberated from the cereal grains and dissolved in water [8]. *This suggests that the mobility of alkylresorcinols in such a water environment is very limited.*

2) **ARs would be detected on (and indeed, likely concentrated on) the outer surfaces of the sherd.** If the source for the alkylresorcinols in our samples were to be the sediments or the water body itself, a passive absorption of ARs would be expected with highest concentration of ARs found on the outer parts of the sherd. On the other hand, it is well known from reference experiments that cooking of lipid-rich resources can lead to high quantities of lipids being absorbed into the inner parts of the ceramics, with highest concentrations potentially towards the inside wall of the pot [11]. We tested this hypothesis here by the analysis of the lipids extracted from the surfaces of three sherds from Loch Bhorgastail, which already showed detectable quantities of ARs in the absorbed residues. In contrast *ARs were not detected on the exterior (inner, nor outer) surfaces of any of these sherds.*

3) **ARs would either be present in every sherd, or show spatial patterning associated with burial at a particular discrete location.** In case of an environmental source of ARs, they would be expected to be present in all samples from a given site, or show spatial patterning within a site, given the close temporal and spatial association between the ceramic samples. However, *ARs were in fact only present in approximately 30% of sherds analysed and there is no spatial clustering in the locations of these sherds containing ARs (**Supplementary Figure 6**). Furthermore, ARs were present in only some samples and not others found within the same context and thus very close spatial proximity.*

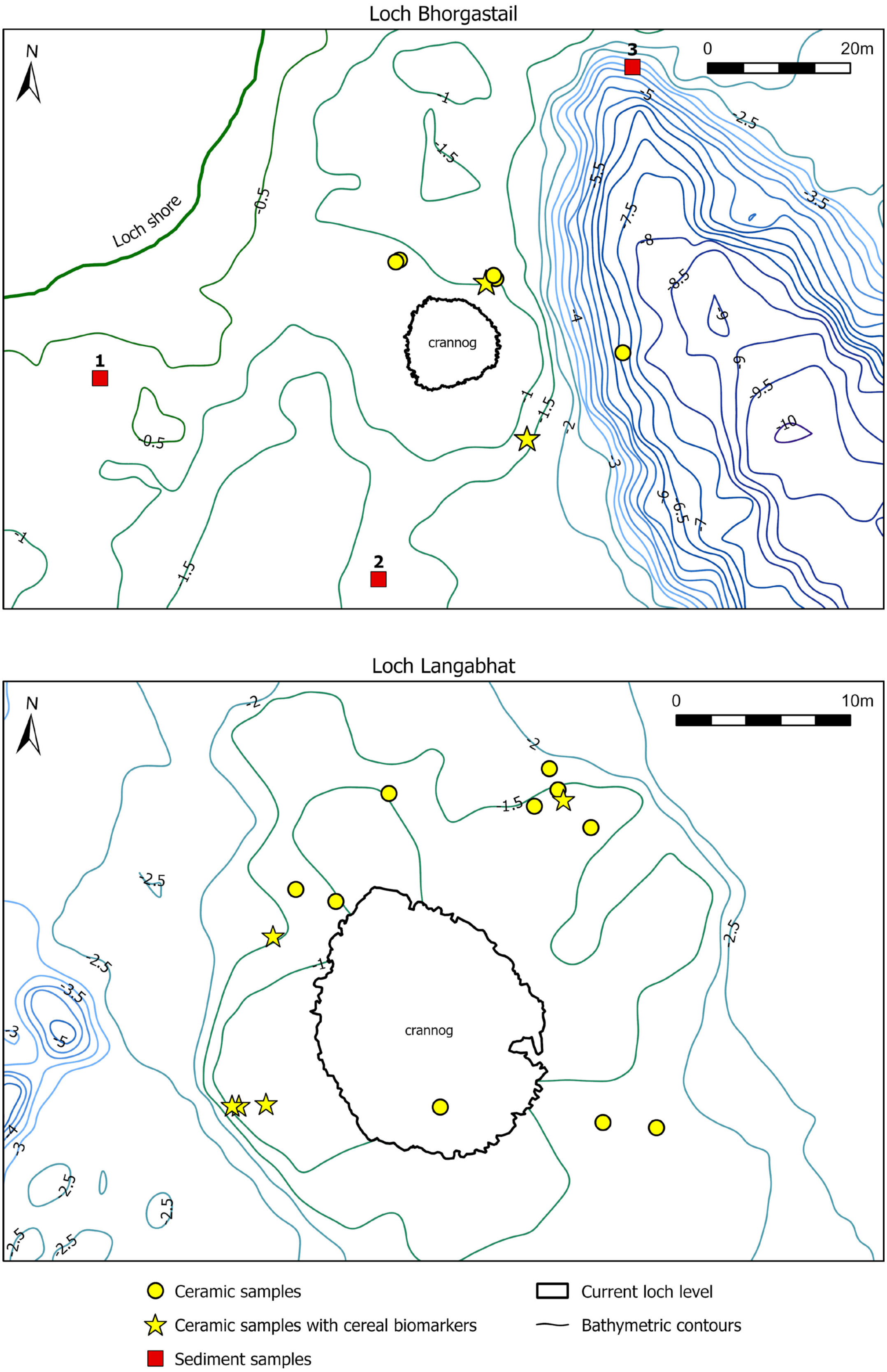

Supplementary Figure 6: Maps of Loch Bhorgastail and Loch Langabhat. The maps show the positions of ceramic samples analysed (yellow circles), samples with detectable quantities of cereal biomarkers (yellow stars) and sediment samples collected at Loch Bhorgastail (red squares). Please note, that some positions indicate more than one sherd found within the same context.

4) Patterns of plant-derived biomarkers would be homogenous and not bear any patterning with ancient anthropogenic activity. Similar to alkylresorcinols, other environmentally derived lipid compounds would be expected to be absorbed in the ceramic sherds. In this case, for a given site a homogenous distribution of these compounds would be expected, as all were exposed to the same environmental conditions. Testing this for long chain alcohols and fatty acids, it is apparent that this is not the case in our samples where we have stark differences in terms of predominating alcohol homologue and general pattern (**Supplementary Figure 7+8**).

Furthermore, we observed a clear correlation of ARs with smaller vessels, as well as a co-occurrence with dairy lipids (see **Figures 5 and 6**). This means that their presence in pots is not random but follows a material culturally-associated pattern, and, therefore followed a specific pattern of use which is not explainable through environmental uptake.

This suggests that these cereal lipids were not absorbed through the burial environment but reflect different resource input into the individual pots and thus a clear anthropogenic signal.

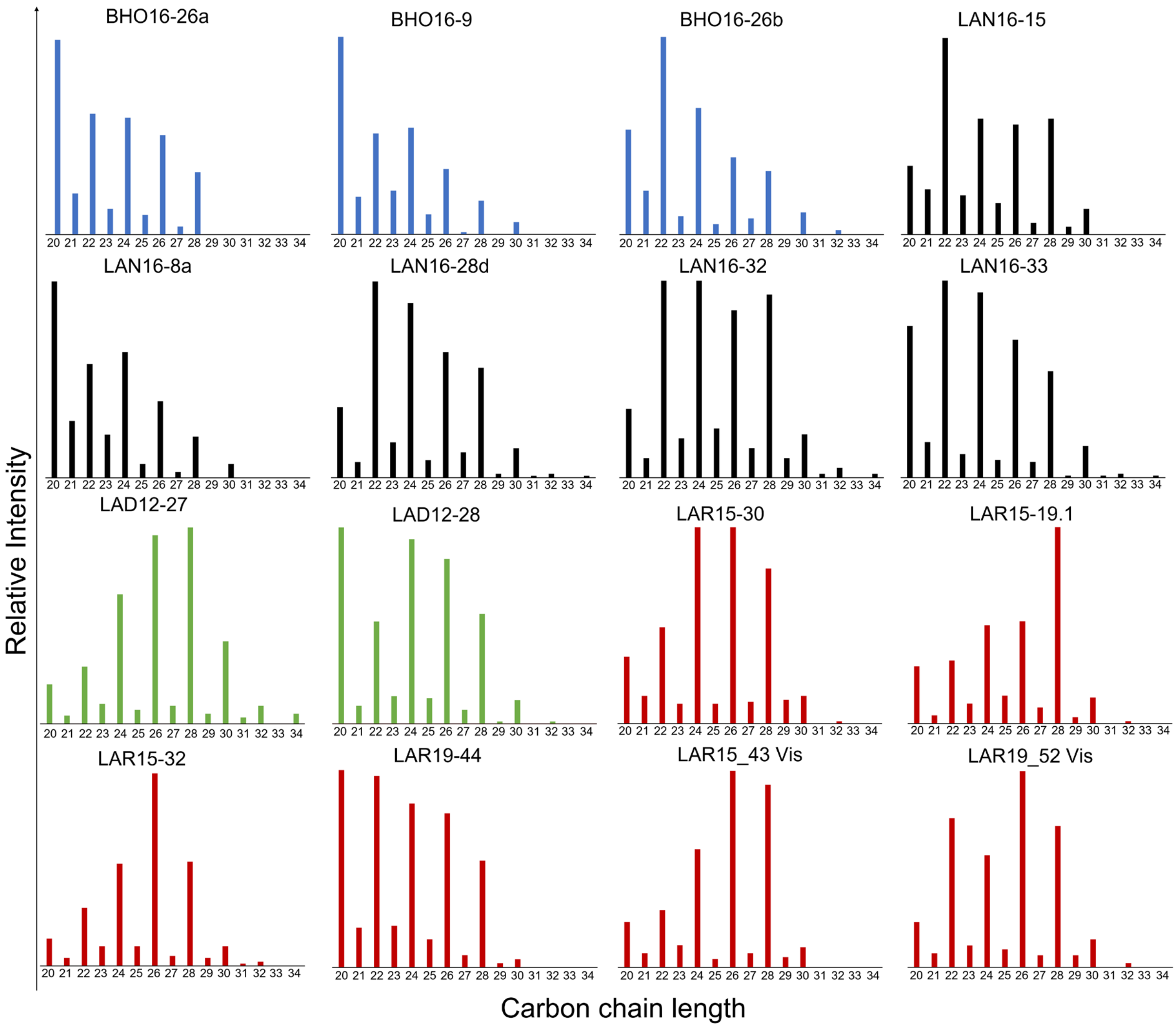

Supplementary Figure 7: Bar charts showing the distributions of C_{20}-C_{34} *n*-alcohols in lipid extracts from sherds that contained alkylresorcinols. Samples from Loch Bhorgastail (BHO), Loch Langabhat (LAN), Loch an Duna (LAD) and Loch Arnish (LAR) are displayed in blue, black, green and red, respectively, determined through GC-QToF MS analysis. The bar charts are normalized to the most abundant homologue in each sample. Source data are provided as a Source Data file.

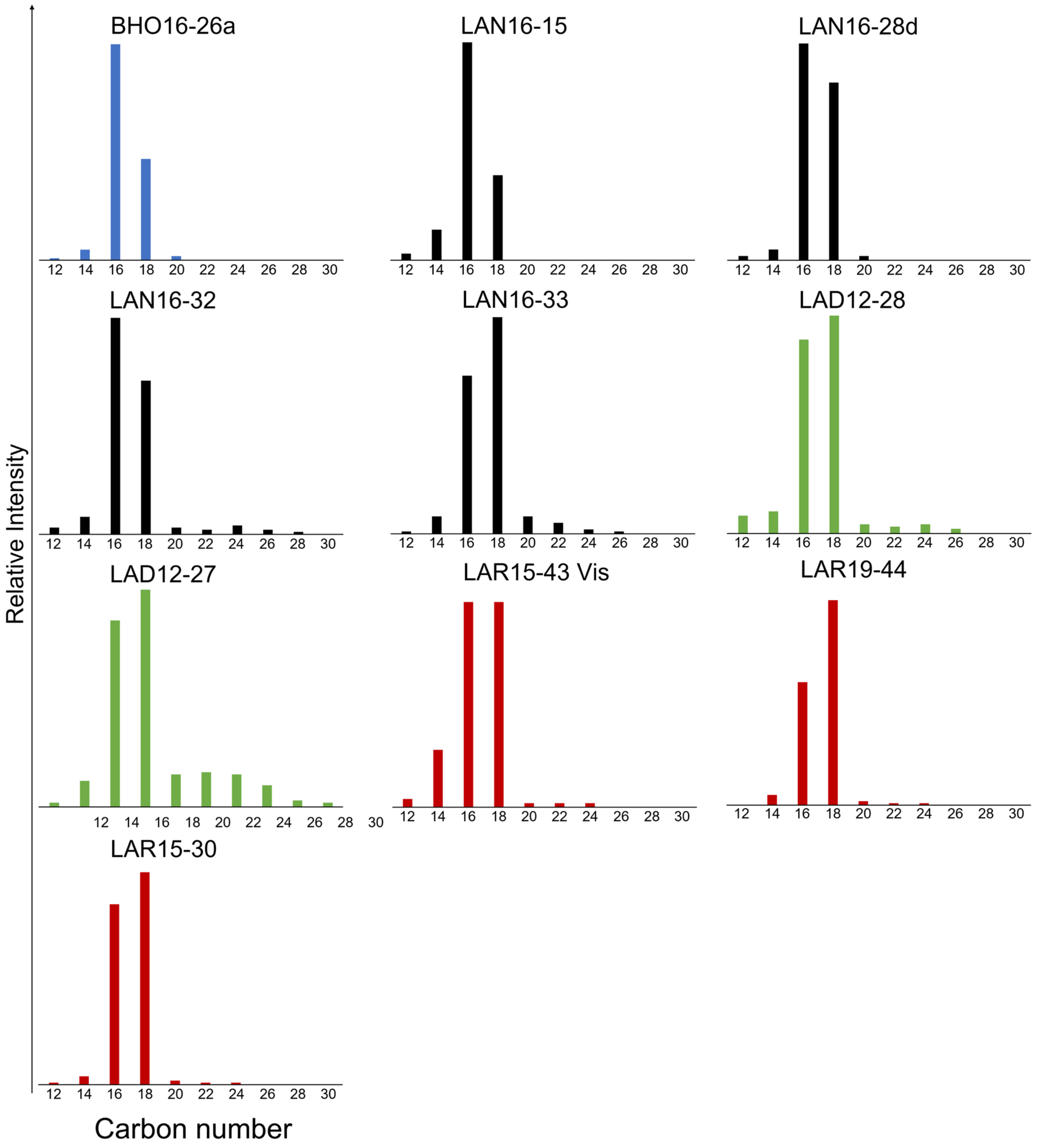

Supplementary Figure 8: Bar charts showing the distributions of C_{12}-C_{30} fatty acids in lipid extracts from sherds which contained alkylresorcinols. Samples from Loch Bhorgastail (BHO), Loch Langabhat (LAN), Loch an Duna (LAD) and Loch Arnish (LAR) are displayed in blue, black, green and red, respectively, determined through GC-QToF MS analysis. The bar charts are normalized to the most abundant homologue in each sample. Only samples could be taken into account which were analysed by GC-MS without prior SPE enrichment step (see Table 1), since in this step the free fatty acids were separated and discarded. Source data are provided as a Source Data file.

5) Finger-prints of plant-derived biomarkers in pottery residues would match those in burial sediments. Ongoing excavations at Loch Bhorgastail allowed us to collect several sediment samples at this site to compare the composition of lipid biomarkers with our samples from this site, in which alkylresorcinols were detected, i.e. BHO16-9, BHO16-26a and BHO16-26b.

In all three sediment samples alkylresorcinols and plant sterols could be detected, with samples 1 and 2 only showing very low abundant peaks of AR-21 and AR-27 while sample 3 showed a wider distribution of ARs. In all three sediment samples long chain alkylresorcinol homologues (AR-25 or longer) were detected, which were completely absent in our samples (**Supplementary Figure 9+10)**. These would be expected to be also present in the archaeological residues in case of an environmental input, but were not.

Sediment sample 3 was vastly more rich in alkylresorcinols and other organic material, but was collected in deeper parts of the loch bed compared to the other two sediment samples and the ceramic samples (**Supplementary Figure 6**). Noteworthy, in one ceramic sample found in depths similar to sediment sample 3 no cereal biomarkers were detected, thus showing no relationship between lipid composition of sediments and ceramic samples.

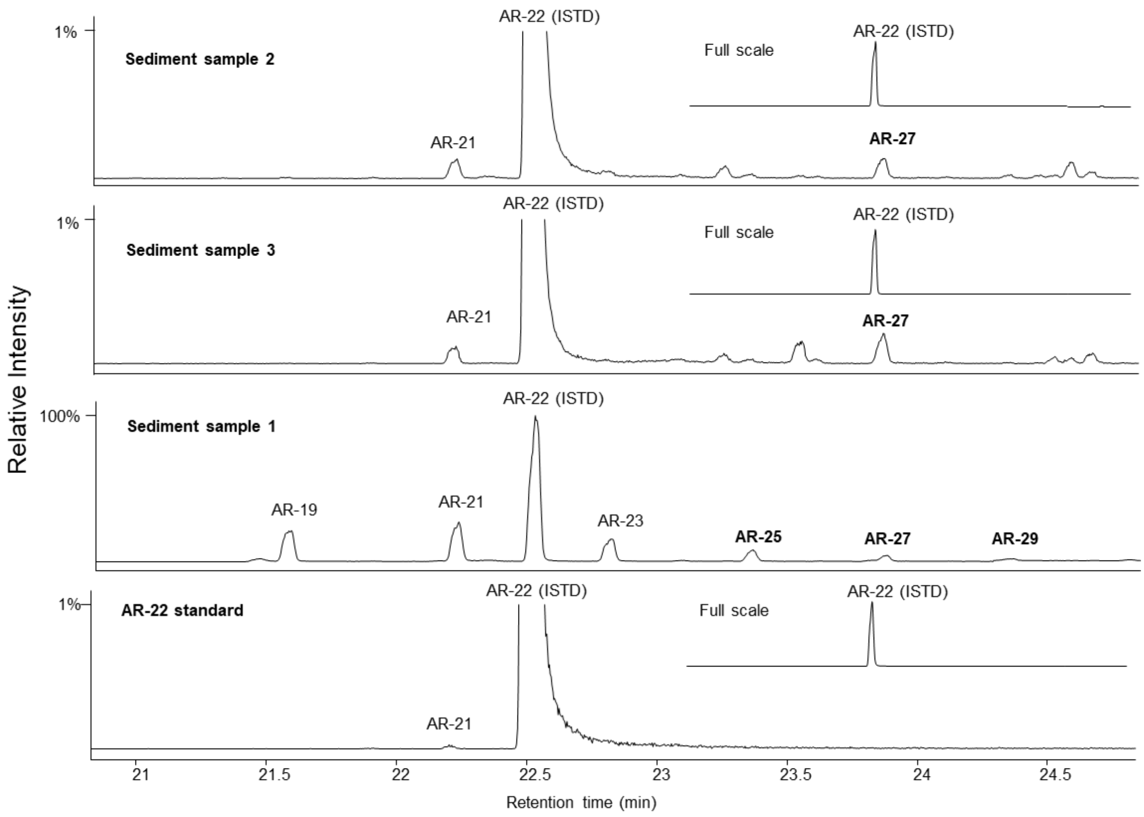

Supplementary Figure 9: Partial GC-QToF MS extracted ion chromatograms (m/z 268.1315) of the three sediment samples from Loch Bhorgastail as well as the AR-22 standard showing the elution of alkylresorcinols (denoted as AR-X with X carbons in the alkyl chains). Sediment sample 3 is show in full scale and sediment samples 1 and 2 and the AR-22 standard are zoomed into to ca. 1% relative intensity. Small inserts on the right side show the full scale chromatogram. Please note, that a low contamination of AR-21 was found in the neat AR-22 standard (ca. 0.05%), which is **not** responsible for the presence of AR-21 in the samples, where a significantly lower quantity was used. Peaks marked in bold were **not** detected in any ceramic samples.

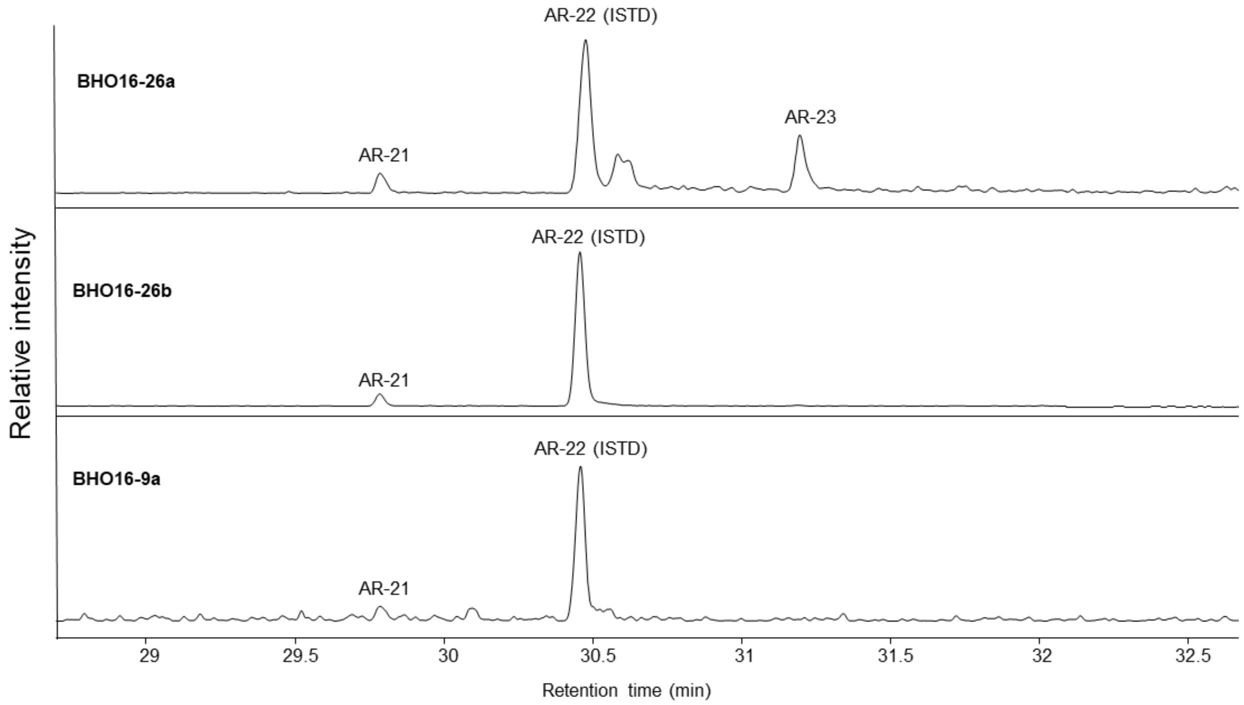

Supplementary Figure 10: Partial GC-QToF MS extracted ion chromatograms (m/z 268.1315, smoothed) of extracts of absorbed residues from samples BHO16-26a, BHO16-26b, and BHO16-9a showing the elution of alkyresorcinols (denoted as AR-X with X carbons in the alkyl chains) AR-21 and AR-23 as well as the internal standard (AR-22)

Furthermore, the patterns of n-alcohols were inconsistent between extracts from the ceramic samples and the sediment samples: While in the ceramic samples C_{20} or C_{22} alcohols were most abundant, highest abundances were found at C_{26} or C_{28} in the sediment samples (**Supplementary Figure 11**). *This again speaks against a general uptake of environmental lipids into the ceramics.*

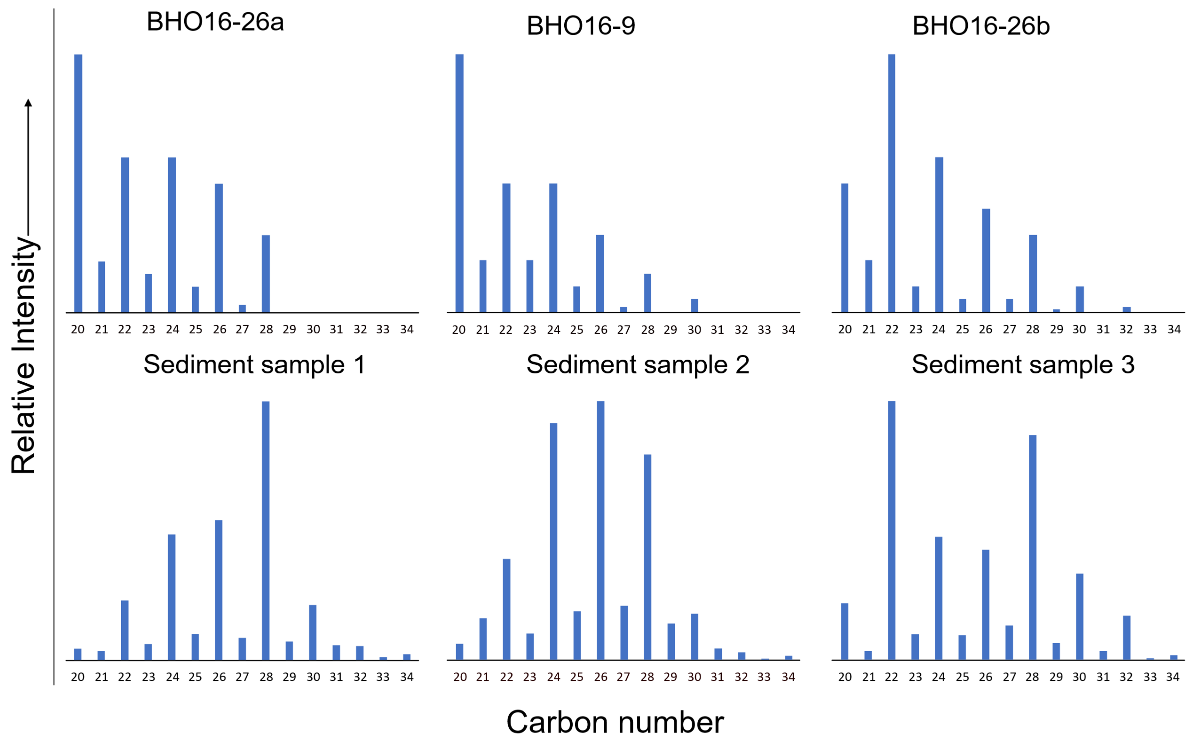

Supplementary Figure 11: Bar charts showing the distributions of C_{20}-C_{34} *n*-alcohols in lipid extracts from sherds that contained alkylresorcinols and three sediment samples from Loch Bhorgastail (BHO), determined through GC-QToF MS analysis. The bar charts are normalized to the most abundant homologue in each sample. Source data are provided as a Source Data file.

6) Finger-prints of plant-derived biomarkers in pottery residues would match published compositions in peat and sedges.

Alkylresorcinols (and plant sterols) have also been detected in sedges and organic matter of peat formed from these [9,10]. In the plants AR-21 was found to be the most abundant homologue (55% of total ARs) with further contributions of AR-23 (20%), AR-19 (16%) and AR-25 (7%). In the peat either AR-21 or AR-19 was found as the most abundant alkylresorcinol homologue depending on sample depth [10], while our pottery samples consistently featured AR-21 as main homologue and AR-19 and AR-23 were only detectable in a low number of pottery lipid extracts.

In the sedge-derived peat *n*-alkanols from C_{20} to C_{34} were reported with generally low abundant C_{20} homologue and a predominating C_{22} homologue, though this depended on the sample depth [9]. In contrast, in the majority of our ceramic samples which contained ARs the C_{20} alcohol had a relative intensity of at least 20% and indeed, in seven samples it was the most abundant alcohol (**Supplementary Figure 7**). Furthermore, in extracts from our pottery samples the C_{32} and C_{34} alcohols were only

present in traces or were not detected at all, while these were found to be abundant in peat [9]. Similarly, carboxylic acids in peat were reported to maximise at C_{24} or C_{26} [9]. In our samples, long chain fatty acids were generally only low abundant compared to the C_{16} and C_{18} fatty acids (**Supplementary Figure 8**). While this is common for mixtures of degraded animal fats with plant-derived lipids there was no obvious bimodal distribution maximising both at C_{16}/C_{18} and C_{24}/C_{26} which would indicate significant input of plant-derived fatty acids

Therefore, AR and particularly n-alcohol and fatty acid patterns identified in the pottery residues do not match the fingerprints identified in published compositions of sedges and sedge derived peats.

With these observations in mind we therefore consider an environmental source for the ARs found in our samples highly unlikely, but the evidence strongly suggests that this represents a genuine food-related signal.

Supplementary Tables:

Supplementary Table 1: **Radiocarbon dates** obtained so far from Loch Arnish, Loch an Duna (Ranish) Loch Bhorgastail and Loch Langabhat. Radiocarbon ages calibrated to the calendar timescale using OxCal 4.3.2[12]. Date ranges calibrated using the IntCal13 atmospheric calibration curve [13]. Note the previous publications of the OxA-coded dates [14] and SUERC-coded dates [15]

**Lab code**	**Site name**	**Material**	**Species/type**	**Context**	**Radiocarbon age**	**δ13C (‰) (error ± 0.2 per mille)**	**Calibrated date range (cal BC) at 95% confidence**
OxA-28953	Loch Arnish	Charred residue	Internal food residue on 'Hebridean' vessel	Unstratified - loch bed	4620±30	-26.3	3510-3350
OxA-28955	Loch an Duna (Ranish)	Charred residue	Internal food residue on 'Hebridean' vessel	Unstratified - loch bed	4658±30	-26.3	3520-3370
OxA-28954	Loch Bhorgastail	Charred residue	Internal food residue on 'Hebridean' vessel	Unstratified - loch bed	4749±30	-21.6	3640-3380
SUERC-77427	Loch Bhorgastail	Wood	Salix sp. - outer rings	[30] - worked timbers east of islet	4737±24	-27.3	3630-3380
SUERC-77428	Loch Bhorgastail	Wood	Salix sp. - outer rings	[31] - worked timbers east of islet	4629±23	-27.4	3500-3360
SUERC-77434	Loch Langabhat	Charred residue	Internal food residue on 'Hebridean' vessel	Unstratified - loch bed, findspot [8]	4708±25	-26.4	3630-3380
SUERC-77432	Loch Langabhat	Wood charcoal	Alnus cf glut - small-medium branch	[52] - occupation deposits inside structure F1	3089±24	-25.4	1420-1290
SUERC-77433	Loch Langabhat	Wood charcoal	Alnus cf glut - medium branch	[57] - occupation deposits inside structure F1	2996±24	-26.2	1370-1130

Supplementary Table 2: **Results from organic residue analysis of the ceramic sherds.** Lipid contents, $\delta^{13}C$ and $\Delta^{13}C$ values for 16:0 and 18:0 fatty acids ($\Delta^{13}C = \delta^{13}C_{18:0} - \delta^{13}C_{16:0}$), other notable lipids, and assignment of lipid sources in the analysed sherds. For interpretation lipid extracts with $\Delta^{13}C$ values < -3.5 ‰ were considered to be pure dairy lipids. Abbreviations: AR – alkylresorcinol, TG – triacylglycerol. LAD = Loch an Duna (Ranish), LAR = Loch Arnish, BHO = Loch Bhorgastail, LAN = Loch Langabhat. Relative vessel sizes are estimates based upon rim diameter, sherd thickness and vessel form.

Sample	Vessel type	Relative vessel size	Lipid content (µg/g)	$\delta^{13}C_{16:0}$ (‰)	$\delta^{13}C_{18:0}$ (‰)	$\Delta^{13}C$ (‰)	Interpretation based on $\delta^{13}C$ analysis	Other biomarkers present	Comment
LAD12-21	Unknown	unknown	<5	-	-	-	-	-	-
LAD12-22	Ridged baggy jar	Large	324	-28.8	-32.0	-3.2	Mixture of dairy and ruminant carcass fat	TG C_{46}-C_{54}, plant sterols	Analysed by GC-MS after further SPE clean-up
LAD12-23	Baggy jar	Large	24	-27.1	-30.1	-3.0	Mixture of dairy and ruminant carcass fat	TG C_{44}-C_{54}, plant sterols	-
LAD12-27	Necked jar	Medium	37	-28.2	-33.3	-5.1	Dairy fat	Plant sterols, AR-21	-
LAD12-28	Ridged baggy jar	Large	<5	-	-	-	-	Plant sterols, AR-21	-
LAD12-29	Ridged baggy jar	Large	125	-28.1	-33.0	-4.9	Dairy fat	TG C_{48}-C_{54}, Ketones C_{31}, C_{33}, C_{35}	-
LAD12-31	Globular vessel	unknown	<5	-	-	-	-	-	-
LAD13-1	Jar	unknown	269	-28.5	-31.4	-2.8	Mixture of dairy and ruminant carcass fat	TG C_{46}-C_{54}, plant sterols,	Analysed by GC-MS after further SPE clean-up
LAD13-2	Ridged baggy jar	Large	230	-28.3	-31.4	-3.1	Mixture of dairy and ruminant carcass fat	TG C_{46}-C_{54}, plant sterols,	Analysed by GC-MS after further SPE clean-up
LAD13-3	Jar	Medium	124	-28.1	-31.6	-3.5	Mixture of dairy and ruminant carcass fat	TG C_{46}-C_{54}, Ketones C_{31}, C_{33}, C_{35}, plant sterols	-

LAD13-5	Baggy jar	unknown	<5	-	-	-	-	-	-
LAR12-14	Unknown	unknown	273	-28.2	-31.3	-3.1	Mixture of dairy and ruminant carcass fat	TG C_{48}-C_{54}, plant sterols	-
LAR12-20	Jar?	unknown	<5	-	-	-	-	-	-
LAR12-21	Jar?	Large	671	-27.3	-30.7	-3.4	Mixture of dairy and ruminant carcass fat	TG C_{48}-C_{54}, Ketones C_{31}, C_{33}, C_{35}, plant sterols	
LAR12-56	Baggy jar	unknown	<5	-	-	-	-	-	-
LAR15-3.3	Baggy jar	unknown	<5	-	-	-	-	-	-
LAR15-14.1	Small jar	Small-medium	231	-28.5	-32.8	-4.3	Dairy fat	TG C_{46}-C_{54}, plant sterols	-
LAR15-19.1	Unknown	unknown	227	-27.2	-32.6	-5.4	Dairy fat	TG C_{42}-C_{54}, AR-21, plant sterols	Analysed by GC-MS after further SPE clean-up
LAR15-30	Ridged baggy jar	Large	12	-	-	-	-	Plant sterols, AR-21	-
LAR15-31	Ridged baggy jar	Large	678	-28.2	-31.3	-3.1	Mixture of dairy and ruminant carcass fat	TG C_{46}-C_{54}, plant sterols	Analysed by GC-MS after further SPE clean-up
LAR15-32	Baggy jar	Medium-large	389	-29.2	-33.8	-4.6	Dairy fat	TG C_{46}-C_{54}, plant sterols, AR-21	Analysed by GC-MS after further SPE clean-up
LAR15-43	Jar	Medium	11	-	-	-	-	Plant sterols	-
LAR15-43 Visible Residue	Jar	Medium	431	-	-	-	-	TG C_{46}-C_{54}, plant sterols, AR-21	-
LAR19-44	Ridged baggy jar	Large	<5	-	-	-	-	Plant sterols, AR-21	-
LAR19-46	Shouldered	Medium	471	-28.8	-33.7	-4.9	Dairy fat	TG C_{42}-C_{54}	Analysed by

	bowl								GC-MS after further SPE clean-up
LAR19-47	Ridged baggy jar	Large	229	-28.2	-31.5	-3.3	Mixture of dairy and ruminant carcass fat	TG C_{48}-C_{54}, plant sterols	-
LAR19-48	Jar	unknown	<5	-	-	-	-	-	-
LAR19-51	Ridged baggy jar	Medium-large	46	-	-	-	-	TG C_{46}-C_{54}, plant sterols	Analysed by GC-MS after further SPE clean-up
LAR19-52	Jar	unknown	66	-28.6	-33.8	-5.2	Dairy fat	TG C_{40}-C_{54}, plant sterols	Analysed by GC-MS after further SPE clean-up
LAR19-52 Visible Residue	Jar	unknown	6617	-28.8	-34.2	-5.4	Dairy fat	TG C_{40}-C_{54}, plant sterols, AR-21	Analysed by GC-MS after further SPE clean-up
BHO16-8	Unstan-type bowl	Small	105	-28.5	-33.4	-4.8	Dairy fat	TG C_{44}-C_{54}	-
BHO16-9	Ridged baggy jar	Large	151	-27.9	-33.1	-5.2	Dairy fat	Plant sterols, AR-21 (trace), TG C_{42}-C_{54}	Analysed by GC-MS after further SPE clean-up
BHO16-12a	Ridged baggy jar	Large	143	-27.4	-32.1	-4.7	Dairy fat	Plant sterols, TG C_{42}-C_{54}	Analysed by GC-MS after further SPE clean-up
BHO16-12e	Ridged baggy jar	Large	12	-	-	-	-	plant sterols	Analysed by GC-MS after further SPE clean-up
BHO16-13-1a	Baggy jar	unknown	13	-	-	-	-	Ketones (C_{31}, C_{33}, C_{35})	-
BHO16-13-1b	Baggy jar	unknown	<5	-	-	-	-	Plant sterols	-

BHO16-13-2	Baggy jar	unknown	<5	-	-	-	-	plant sterols	-
BHO16-26a	Unknown	unknown	<5	-	-	-	-	plant sterols, AR-21 (trace)	-
BHO16-26b	Ridged baggy jar	unknown	1221	-27.1	-29.6	-2.6	Ruminant carcass fat	Plant sterols, AR-21 (trace), TG C_{44}-C_{54}, Ketones (C_{31}, C_{33}, C_{35})	Analysed by GC-MS after further SPE clean-up
BHO16-28	Ridged baggy jar	Large	275	-28.4	-31.0	-2.6	Ruminant carcass fat	TG C_{48}-C_{54}	Analysed by GC-MS after further SPE clean-up
BHO17-29b	Ridged baggy jar	Large	<5	-	-	-	-	-	-
BHO17-29d	Ridged baggy jar)	Large	<5	-	-	-	-	Plant sterols	-
LAN16-2	Baggy jar	Large	255	-27.4	-29.7	-2.2	Ruminant carcass fat	Plant sterols, TG C_{46}-C_{54}	Analysed by GC-MS after further SPE clean-up
LAN16-6	Unstan-type bowl	Small	878	-27.4	-33.4	-6.0	Dairy fat	Plant sterols, TG C_{42}-C_{54}	Analysed by GC-MS after further SPE clean-up
LAN16-7b	Ridged baggy jar	Large	15	-	-	-	-	Ketones (C_{31}, C_{33}, C_{35})	
LAN16-8a	Baggy jar	Large	344	-27.4	-33.2	-5.8	Dairy fat	Plant sterols, AR-21, AR-23, TG C_{40}-C_{54}	Analysed by GC-MS after further SPE clean-up
LAN16-13	Baggy jar	Large	<5	-	-	-	-	Plant sterols	-
LAN16-14	Unstan-type bowl	Small	<5	-	-	-	-	Plant sterols	-
LAN16-15	Ridged baggy jar	Large	10	-25.3	-31.4	-6.1	Dairy fat	Plant sterols, AR-21, AR-23	-

LAN16-19	Ridged baggy jar	Medium	89	-26.6	-32.9	-6.3	Dairy fat	Plant sterols, TG C_{44}-C_{54}	Analysed by GC-MS after further SPE clean-up
LAN16-28a	Unstan-type bowl	Small	<5	-	-	-	-	-	-
LAN16-28d	Ridged baggy jar	Large	<5	-	-	-	-	Plant sterols, AR-21	-
LAN16-32	Baggy jar	Large	<5	-	-	-	-	Plant sterols, AR-19, AR-21, AR-23	-
LAN16-33	Ridged baggy jar	Large	24	-27.5	-32.6	-5.1	Dairy fat	Plant sterols, AR-21, AR-23, Ketones (C_{29}, C_{31}, C_{33}, C_{35}, C_{37})	-
LAN16-43	Necked Jar	Small-medium	<5	-	-	-	-	-	-
LAN16-44	Unstan-type bowl	Small	<5	-	-	-	-	Plant sterols	-
LAN16-45	Ridged baggy jar	unknown	<5	-	-	-	-	-	-
LAN16-46	Unstan-type bowl	Small	<5	-	-	-	-	Plant sterols	-
LAN17-50a	Bowl	Small	139	-28.0	-33.6	-5.6	Dairy fat	Plant sterols	-
LAN17-60	Ridged baggy jar	Large	<5	-	-	-	-	-	-
LAN17-61	Jar or bowl	Medium-large	<5	-	-	-	-	Plant sterols	-

Supplementary references

1 Copley, M. S. *et al.* Direct chemical evidence for widespread dairying in prehistoric Britain. *Proc. Natl. Acad. Sci. U.S.A.* **100**, 1524-1529 (2003).

2 Friedli, H., Lötscher, H., Oeschger, H., Siegenthaler, U. & Stauffer, B. Ice core record of the 13C/12C ratio of atmospheric CO2 in the past two centuries. *Nature* **324**, 237 (1986).

3 Cramp, L. & Evershed, R. P. Reconstructing aquatic resource exploitation in human prehistory using lipid biomarkers and stable isotopes in *Treatise on Geochemistry: Archaeology and Anthropology* (eds H. D. Holland & K. K. Turekian) 319-339 (Elsevier, 2014).

4 Ross, A. B. *et al.* Alkylresorcinols in cereals and cereal products. *J. Agric. Food Chem.* **51**, 4111-4118 (2003)..

5 Ziegler, J. U. *et al.* Alkylresorcinol composition allows the differentiation of Triticum spp. having different degrees of ploidy. *J. Cereal Sci.* **65**, 244-251, (2015).

6 Hammann, S., Korf, A., Bull, I. D., Hayen, H. & Cramp, L. J. E. Lipid profiling and analytical discrimination of seven cereals using high temperature gas chromatography coupled to high resolution quadrupole time-of-flight mass spectrometry. *Food. Chem.* **282**, 27-35 (2019).

7 Colonese, A. C. *et al.* New criteria for the molecular identification of cereal grains associated with archaeological artefacts. *Sci Rep* **7**, 6633 (2017).

8 Hammann, S. & Cramp, L. J. E. Towards the detection of dietary cereal processing through absorbed lipid biomarkers in archaeological pottery. *J. Archaeol. Sci.* **93**, 74-81 (2018).

9 Xie, S. *et al.* Molecular and isotopic stratigraphy in an ombrotrophic mire for paleoclimate reconstruction. *Geochim. Cosmochim. Acta* **68**, 2849-2862 (2004).

10 Avsejs, L. A. *et al.* 5-n-Alkylresorcinols as biomarkers of sedges in an ombrotrophic peat section. *Org. Geochem.* **33**, 861-867 (2002).

11 Hammann, S., Scurr, D. J., Alexander, M. R. & Cramp, L. J. Mechanisms of lipid preservation in archaeological clay ceramics revealed by mass spectrometry imaging. *Proc. Natl. Acad. Sci. U.S.A.* **117**, 14688-14693 (2020).

12 Ramsey, C. B. OxCal 4.3 Available at https://c14.arch.ox.ac.uk/oxcalhelp/hlp_contents.html (2018).

13 Reimer, P. J. *et al.* IntCal13 and Marine13 Radiocarbon Age Calibration Curves 0–50,000 Years cal BP. *Radiocarbon* **55**, 1869-1887 (2013).

14 Garrow, D., Griffiths, S., Anderson-Whymark, H. & Sturt, F. Stepping Stones to the Neolithic? Radiocarbon Dating the Early Neolithic on Islands Within the 'Western Seaways' of Britain. *Proc. Prehist. Soc.* **83**, 97-135 (2017).

15 Garrow, D. & Sturt, F. Neolithic crannogs: rethinking settlement, monumentality and deposition in the Outer Hebrides and beyond. *Antiquity* **93**, 664-684 (2019).
