# Supplementary material for: Neolithic culinary traditions revealed by cereal, milk and meat lipids in pottery from Scottish crannogs
Source: Nat Commun. 2022 Sep 6;13:5045. doi: 10.1038/s41467-022-32286-0 (PMC9448721; doi:10.1038/s41467-022-32286-0)
Supplement: Supplementary file 2 — Reporting Summary [file 41467_2022_32286_MOESM2_ESM.pdf]

nature research

Corresponding author(s): Simon Hammann, Lucy J. E. Cramp, Duncan Garrow, Fraser Sturt

Last updated by author(s): Jun 8, 2022

Reporting Summary

Nature Research wishes to improve the reproducibility of the work that we publish. This form provides structure for consistency and transparency in reporting. For further information on Nature Research policies, see Authors & Referees and the Editorial Policy Checklist .

Statistics

For all statistical analyses, confirm that the following items are present in the figure legend, table legend, main text, or Methods section.

n/a	Confirmed	
☐	☒	The exact sample size (*n*) for each experimental group/condition, given as a discrete number and unit of measurement
☒	☐	A statement on whether measurements were taken from distinct samples or whether the same sample was measured repeatedly
☒	☐	The statistical test(s) used AND whether they are one- or two-sided<br>*Only common tests should be described solely by name; describe more complex techniques in the Methods section.*
☒	☐	A description of all covariates tested
☒	☐	A description of any assumptions or corrections, such as tests of normality and adjustment for multiple comparisons
☐	☒	A full description of the statistical parameters including central tendency (e.g. means) or other basic estimates (e.g. regression coefficient) AND variation (e.g. standard deviation) or associated estimates of uncertainty (e.g. confidence intervals)
☒	☐	For null hypothesis testing, the test statistic (e.g. *F*, *t*, *r*) with confidence intervals, effect sizes, degrees of freedom and *P* value noted<br>*Give P values as exact values whenever suitable.*
☒	☐	For Bayesian analysis, information on the choice of priors and Markov chain Monte Carlo settings
☒	☐	For hierarchical and complex designs, identification of the appropriate level for tests and full reporting of outcomes
☒	☐	Estimates of effect sizes (e.g. Cohen's *d*, Pearson's *r*), indicating how they were calculated

Our web collection on statistics for biologists contains articles on many of the points above.

Software and code

Policy information about availability of computer code

Data collection	GC-FID: Data were collected using Chemstation (version B.03.02, Agilent). GC-QToF MS: Date were collected using MassHunter (version B.07.02.1938. GC-C-IRMS: Data were collected using Ion Vantage version 1.6.1.0 (IsoPrime)
Data analysis	GC-FID: Data analysed using Chemstation (version B.03.02, Agilent). GC-QToF MS: Date were analysed using Qualitative Analysis B.07.00 (Agilent) and MZmine 2.5. GC-C-IRMS: Data processing was carried out using Ion Vantage version 1.6.1.0 (IsoPrime). Radiocarbon: Radiocarbon ages were calibrated using OxCal 4.3.2

For manuscripts utilizing custom algorithms or software that are central to the research but not yet described in published literature, software must be made available to editors/reviewers. We strongly encourage code deposition in a community repository (e.g. GitHub). See the Nature Research guidelines for submitting code & software for further information.

Data

Policy information about availability of data

All manuscripts must include a data availability statement. This statement should provide the following information, where applicable:

- Accession codes, unique identifiers, or web links for publicly available datasets
- A list of figures that have associated raw data
- A description of any restrictions on data availability

All data needed to support the conclusions of the paper are presented in the paper or the Supplementary Material. The raw GC-QToF MS data have been deposited in the Bristol Research Data Repository under following link: https://data.bris.ac.uk/data/dataset/fn4ujbvbe4nr2eji3icdzvp65. Source data for figures are provided with this paper. All ceramic samples analysed in this project are currently stored at the Universities of Reading and Southampton, pending final deposition at the end of the project according to the Scottish Treasure Trove process. For access to the samples please contact D.G. or F.S.

Field-specific reporting

Please select the one below that is the best fit for your research. If you are not sure, read the appropriate sections before making your selection.

- [x] Life sciences
- [ ] Behavioural & social sciences
- [ ] Ecological, evolutionary & environmental sciences

For a reference copy of the document with all sections, see nature.com/documents/nr-reporting-summary-flat.pdf

Life sciences study design

All studies must disclose on these points even when the disclosure is negative.

Sample size	Sample size was largely determined by limited available sample material from the archaeological sites. Sherds from near the rim of the vessel were chosen where possible to maximise the chances to yield sufficient lipids for analysis. We aimed to have at least 10 - 15 samples per site.
Data exclusions	No data were excluded. Samples with insufficient fatty acid contents were not analysed by GC-C-IRMS.
Replication	Samples were analysed once by GC-FID and GC-QToF MS. Since these analyses were only for qualitative and semi-quantitative purposes no replicate analyses were performed. All GC-C-IRMS were injected in duplicate. If individual d13C values deviated by more than 0.5‰ the results were discarded and the analysis was repeated. Deviations in δ13C values between duplicates for all samples were <0.3‰.
Randomization	The samples per site were not extracted or analysed in any specific order. Since detailed inter or intra-site comparisions were not the scope of this study, further randomisation was not required for this study
Blinding	Detailed inter or intra-site comparisions of data from separate sample groups were not part of this study, so blinding of investigators was not required

Reporting for specific materials, systems and methods

We require information from authors about some types of materials, experimental systems and methods used in many studies. Here, indicate whether each material, system or method listed is relevant to your study. If you are not sure if a list item applies to your research, read the appropriate section before selecting a response.

Materials & experimental systems

n/a	Involved in the study	
☒	☐	Antibodies
☒	☐	Eukaryotic cell lines
☒	☐	Palaeontology
☒	☐	Animals and other organisms
☒	☐	Human research participants
☒	☐	Clinical data

Methods

n/a	Involved in the study	
☒	☐	ChIP-seq
☒	☐	Flow cytometry
☒	☐	MRI-based neuroimaging
